# A New Social Conflict on Globalisation-Related Issues in Germany? A Longitudinal Perspective

**DOI:** 10.1007/s11577-023-00884-5

**Published:** 2023-05-08

**Authors:** Céline Teney, Li Kathrin Rupieper

**Affiliations:** 1 Institute of Sociology, Freie Universität Berlin, Garystr. 55, 14195 Berlin, Germany; 2 Institut für Wirtschaftspolitik, Leibniz Universität Hannover, Königsworther Platz 1, 30167 Hannover, Germany

**Keywords:** Globalisation cleavage, Opinion polarisation, Issue salience, Attitudes, German population, Longitudinal analysis, Globalisierungs-Cleavage, Meinungspolarisierung, Themensalienz, Einstellungen, Deutsche Bevölkerung, Längsschnittanalyse

## Abstract

We draw on cleavage theory to assess the emergence of a social conflict concerning globalisation-related issues among the German population between 1989 and 2019. We argue that issue salience and opinion polarisation are key conditions for a successful and sustainable political mobilisation of citizens and thus for the emergence of a social conflict. In line with globalisation cleavage theory, we hypothesised that issue salience as well as overall and between-group opinion polarisation on globalisation-related issues have increased over time. Our study considers four globalisation-related issues: immigration, the European Union (EU), economic liberalism, and the environment. While the salience of the EU and economic liberalism issues remained low during the observed period, we found a recent increase in salience for the issues of immigration (since 2015) and the environment (since 2018). Furthermore, our results point to rather stable attitudes on globalisation-related issues among the German population: We did not find any consistent evidence of an increase in overall or between-group polarisation over time. In conclusion, the idea of an emerging conflict around globalisation-related issues among the German population finds very little empirical support.

## Abstract (de)

Im Rückgriff auf die Cleavage-Literatur untersuchen wir, ob in der deutschen Bevölkerung zwischen 1989 und 2019 ein sozialer Konflikt rund um Globalisierungsthemen entstanden ist. Wir argumentieren, dass sowohl Themensalienz als auch Meinungspolarisierung notwendige Bedingungen für eine erfolgreiche und anhaltende politische Mobilisierung von Bürgerinnen und Bürgern sind, und daher auch für das Entstehen sozialer Konflikte. In Übereinstimmung mit der Globalisierungs-Cleavage-Theorie stellen wir die Hypothesen auf, dass sowohl die Themensalienz von als auch die gesamt- und teilgesellschaftliche Meinungspolarisierung um Globalisierungsthemen über die Zeit gestiegen ist. Unsere Studie berücksichtigt die vier Globalisierungsthemen „Einwanderung“, „EU“, „Wirtschaftsliberalismus“ und „Umwelt“. Während die Salienz der Themen „EU“ und „Wirtschaftsliberalismus“ über den gesamten Beobachtungszeitraum gering bleibt, finden wir kürzliche Anstiege in der Salienz von „Immigration“ (seit 2015) und „Umwelt“ (seit 2018). Außerdem weisen unsere Ergebnisse auf recht stabile Einstellungen zu Globalisierungsthemen in der deutschen Bevölkerung hin: Wir finden keine konsistenten Belege für einen Anstieg der gesamt- oder teilgesellschaftlichen Meinungspolarisierung im Verlauf der Zeit. Abschließend lässt sich also festhalten, dass die Idee eines entstehenden Globalisierungskonfliktes in der deutschen Bevölkerung wenig Unterstützung in der Empirie findet.

## Introduction

In the last decade, consolidated western democracies have been facing increasingly successful political mobilisation against globalisation. Globalisation refers to the widening, deepening, and speeding up of worldwide interconnectedness in its economic, sociocultural, and political dimensions. The 2016 Brexit referendum, the 2016 US presidential election of Trump, and the continuing electoral success of Marine Le Pen from the French National Rally comprise the most prominent examples of such a successful mobilisation. Germany is no exception to this trend: The Alternative for Germany party (AfD) with its anti-immigration and Eurosceptic programme seems to have managed a breakthrough within the German political party landscape. These examples illustrate the rise of a new social conflict around globalisation-related issues. The aim of this article is to investigate empirically the emergence of such a conflict on globalisation-related issues among the German population. To this end, we draw on cleavage theory and consider the positive function social conflicts can have for social integration (see also Deitelhoff and Schmelzle (2023) for a similar perspective). We use survey data covering the past three decades to assess the emergence of such a conflict among the German population. Our longitudinal perspective enables us to contextualise the current and lively public and academic debates on opinion polarisation and conflict around globalisation-related issues. Indeed, understanding the historical evolution of such a conflict can put into perspective the alarmist voices that have become louder in the current academic and public debates on the rise of a new globalisation cleavage.

Our contribution is threefold: First, we shed light on the role of such conflicts for social integration according to cleavage theory and derive hypotheses on the emergence of a conflict on globalisation-related issues at societal and group levels. Second, we provide a conceptualisation of conflict that enables a straightforward empirical assessment of conflict evolution. By mirroring the literature on the politicisation of public debate (e.g., Hutter et al. 2016), we argue that opinion polarisation and issue salience are the two necessary conditions for sustainable political mobilisation—and thus for conflict—among the population. Lastly, we investigate empirically the extent to which a conflict on globalisation-related issues has emerged in Germany by analysing survey data covering the last three decades. To this end, we assess the evolution of issue salience and attitudinal polarisation among the German population since the 1990s in respect of four issues related to globalisation: economic liberalism, the European Union (EU), the environment, and immigration. We selected these four issue domains because they represent different dimensions of the growing interconnectedness between nation states. Economic liberalism refers to the border crossing of goods. The European integration process is an example of border-crossing political authority. Immigration can be defined as the border crossing of people. Lastly, the environmental issue refers to the border crossing of pollutants, with climate change as the currently most prominent example of the global interconnectedness of environmental issues.

In a nutshell, our results point to a recent increase in the salience of “immigration” (since 2015) and “environment” (since 2018) such that by the end of 2019, these issues were among the most frequently identified problems mentioned by Germans. By contrast, the salience of “economic liberalism” and “Europe” remained very low during the observed time period of 1989–2019. Furthermore, our results point to rather stable attitudes on globalisation-related issues among the German population over time: We do not find any consistent evidence of an increase in overall and between-group polarisation in respect of our four globalisation-related issues. All in all, the idea of an emerging conflict around globalisation-related issues among the German population finds very little empirical support.

Our contribution is structured as follows: First, we draw on cleavage theory to describe the emergence of a conflict on globalisation-related issues. We then conceptualise conflict and argue that its emergence has two necessary conditions: issue salience as well as opinion polarisation within the overall population and between groups. When presenting these key conditions of a conflict, we hypothesise that issue salience and overall and between-group opinion polarisation have increased over time with respect to globalisation-related issues. Next, we present the data and methods used in our empirical analysis. This is followed by the presentation and interpretation of our results, and finally, our conclusion.

## The Role of Social Conflict for Social Integration

The recent successful and sustainable political mobilisation against globalisation observed in various consolidated western democracies has been interpreted as the manifestation of a social conflict around globalisation-related issues. According to the landmark work of Kriesi et al. (2008), globalisation pressures lead to the polarisation of citizens into groups of winners and losers who support antagonistic positions on globalisation-related issues: Globalisation’s losers tend to endorse positions favouring more national closure, while winners tend to support positions favouring more transnational integration and denationalisation. Scholars have indeed repeatedly pointed to an attitudinal divide on international trade (Jungherr et al. 2018), EU integration (De Vries 2018), immigration (e.g., Teney et al. 2014), and the fight against climate change (e.g., McCright et al. 2016) among citizens in Europe.

When pointing to the rise of a globalisation cleavage in Western Europe, Kriesi et al. (2008) use the theoretical framework provided by Lipset and Rokkan (1967) in their seminal work on cleavages. Despite their focus on party competition, Lipset and Rokkan (1967) also describe the structuration of the population and its political mobilisation on social conflicts—the demand side of the political system. Focusing on the demand side of cleavage theory can shed light on the function and implications of such structural conflicts for social integration. According to Lipset and Rokkan (1967), the process of nation state formation led to the crystallisation of new conflicts based on opposing interests between citizens living in peripheral communities and supporters of a centralised state, and between secularists and defenders of the church. Furthermore, the Industrial Revolution drove the crystallisation of new interest conflicts between citizens living in rural communities and citizens living in urban communities as well as between workers and employers or owners of the means of production. The process of repeated conflicts with other groups solidified collective identities and in-group solidarity. These social conflicts were thus structuring the society. They became rooted in grassroots movements and hierarchical organisations such as churches and labour unions (Hooghe and Marks 2018). National mass political parties emerged as a channel for the expression and mobilisation of protest. Political parties thus became the instrument of expression of protest movements and “help to crystallize and make explicit the conflicting interests, the latent strains and contrasts in the existing social structure” (Lipset and Rokkan 1967, p. 5).

Lipset and Rokkan (1967) argue that these fundamental societal divisions lead to the emergence of sustainable sociocultural cleavages that structure party competition. Accordingly, external shocks such as the Industrial Revolution lead to the rise of new conflicts between social groups that become rooted and organisationally institutionalised. These institutionalised social conflicts—or sociocultural cleavages—constitute the way societies are structured in periods of relative stability. They themselves become a means of political stabilisation by providing individuals with a constellation of preexisting alternatives for their own social and political integration (Bartolini 2000, p. 22). In periods of abrupt changes resulting from external shocks, new social conflicts emerge and coexist with the prior cleavages. They force political parties to adjust to the realignment of citizens along the new conflict lines. This adjustment can take the form of intense internal friction within existing parties or in challenging parties focusing on the new conflict lines that emerge alongside the established parties (Hooghe and Marks 2018).

Globalisation pressures—in particular with the opening up of national borders to goods with international trade, to people with immigration, and to a supranational authority with the EU—constitute such an external shock that the result is the rise of new conflict lines between social groups (Kriesi et al. 2008). According to cleavage theory, until recently we have been experiencing a period of abrupt change with increasing globalisation pressures (Hooghe and Marks 2018). This period is characterised by the emergence of new lines of social conflicts around globalisation-related issues. This, in turn, adds a further form of sense of belonging and in-group solidarity to this multilayered structure of collective identities. These new group boundaries are built on contrasting levels of human capital and cosmopolitan or national outlook (Bornschier et al. 2021). Political parties need to adjust to this realignment of citizens showing conflictual interests on globalisation-related issues. This period of abrupt change can also lead to the emergence of challenging parties seeking to mobilise citizens on these globalisation-related issues, such as the AfD in Germany or the UK Independence Party (UKIP) in the United Kingdom. Once the political system has adjusted to this new social conflict, we will accordingly face a period of relative stability within society, as political parties will provide a channel for the expression of conflicting interests on globalisation-related issues.

According to cleavage theory, a conflict on globalisation-related issues has an integrative function by providing citizens with collective identities, in-group solidarity, and preexisting political alternatives. Social integration manifested through political stability is thus closely intertwined with the rooting of social conflicts within society. Both cleavage theory and conflict theory (presented at length in the article by Deitelhoff and Schmelzle, this issue) consider the rooting of social conflicts within society to be an essential element of social integration. A major difference between these theories is Parsons’s structural–functional framework used by Lipset and Rokkan in their cleavage theory: They assume that political stability will be the outcome of the crystallisation of social conflicts and that cleavages, once arisen and institutionalised, are “frozen” within the society. By contrast, conflict theory (Coser 1956; Dahrendorf 1971; Simmel 1992) aims to unpack the role of social conflicts in societal dynamics and changes. Accordingly, as long as conflict partners respect the common rules and norms of the social structure, conflict contributes to social integration because it requires interactions between actors and implies the building of groups (Bonacker 2003).

The recent theoretical developments of the cleavage literature surrounding the emergence of a globalisation cleavage (e.g., Hooghe and Marks 2018; Kriesi et al. 2008) provide us with a fruitful framework for developing a set of hypotheses on the emergence of a conflict on globalisation-related issues. Indeed, as globalisation pressures have increased continuously in the last decades,^1^ we can expect such a conflict to have emerged in Germany. At this stage we should mention a limitation of our empirical analysis, however: Owing to data availability restrictions, we focus exclusively on the structural component of a cleavage. According to Bartolini and Mair (1990), a cleavage requires three key components. The structural component refers to a coherent and consistent opinion divide within the population on a set of related issues. The organisational component relates to organisations such as political parties or civil society movements that represent the conflicting interests of the population on this set of related issues. Lastly, the normative component is constituted of coherent ideologies and collective identities around the two poles of the sociopolitical divide. In this article, we restrict our empirical assessment of the rise of a new conflict on globalisation-related issues in Germany to the structural component, leaving aside collective identities, common ideologies, and political organisations. Hence, we refer to “conflict” instead of “cleavage” throughout the paper.

## Conceptualisation of Conflict

### Salience and Polarisation as Conditions of Conflict

Two conditions must be met for a political mobilisation based on globalisation-related issues among citizens to be sustainable, and thus to be able to speak of a social conflict. First, we need to observe opinion polarisation on globalisation-related issues. We understand opinion polarisation as a process in which citizens position themselves increasingly on the two polar edges of an attitudinal divide (Barber and McCarty 2015, p. 24).^2^ This characterisation of polarisation as a process is essential for assessing the emergence of a social conflict resulting from increasing globalisation pressures, as suggested by the cleavage literature. Opinion polarisation does indeed result in a decrease in social and political stability, as it reduces the probability of group formation at the centre of the opinion distribution and increases the likelihood of the formation of groups with distinctive, irreconcilable policy preferences (DiMaggio et al. 1996, p. 603). Studies on public opinion on globalisation-related issues based on a single-wave data design have been burgeoning. However, using cross-sectional analyses to point to antagonistic public opinion cannot provide us with information on a potential opinion shift. Furthermore, empirical studies assessing the trend in support of or opposition to a globalisation-related issue have long been established in the social sciences debate (e.g., Czymara and Dochow 2018; Kuhn et al. 2016). However, focusing on the evolution of one polar edge in an attitude divide cannot shed light on shifts in the overall opinion distribution. An adequate measurement of polarisation does indeed require us to consider the entire distribution of an attitudinal item—including both polar edges and the middle-range positions. Longitudinal analyses on the evolution of opinion polarisation on globalisation-related issues in Europe have been much sparser and point to mixed evidence. For instance, Jennings and Stoker (2016) highlight an increase in opinion polarisation on immigration and the EU in the last decades in the United Kingdom. By contrast, other scholars point either to a stable level of polarised opinion or to a depolarisation trend (Munzert and Bauer 2013) or to ambivalent results varying along the attitudinal dimensions surveyed (De Vries et al. 2021).

The fact that a large portion of the population holds antagonistic positions on globalisation-related issues is a necessary but not sufficient condition of a social conflict. Issue salience among the population constitutes the second condition of social conflict. Indeed, polarised public opinion on nonsalient issues is unlikely to lead to a social conflict or to political mobilisation around these issues (Hetherington 2009). Dennison and Geddes (2019) argue, for instance, that the recent and growing success of anti-immigrant right-wing populist parties in Europe is mostly due to an increase in the salience of the immigration issue among the European population during the last decade. Indeed, they show that the proportion of citizens holding anti-immigrant attitudes has remained stable over the last decade, while Europeans seem to have considered immigration as an increasingly important issue over the last decade.

Meanwhile, there is an established research tradition in assessing the salience of globalisation-related issues in national parliaments (Rauh 2015), in the media (Hutter et al. 2016), and in civil society (Meunier and Czesana 2019). However, our knowledge of the evolution of issue salience on globalisation-related topics among the population is much more limited. Indeed, we know only that the salience of immigration among the population has increased in Western Europe in the last decade (Claassen and McLaren 2020; Dennison and Geddes 2019). We will therefore expand this strand of research by assessing the salience of four issue domains related to globalisation: economic liberalism, the EU, immigration, and the environment. We follow the usual operationalisation of issue salience (Dennison 2019) by using an open survey question on the most important problem faced by the respondents’ country.

In sum, by considering opinion polarisation and issue salience as the two conditions of social conflict, we can derive two hypotheses for assessing the emergence of a conflict on globalisation-related issues in Germany:

#### H 1

The salience of the issues of economic liberalism, the EU, immigration, and the environment has increased over time among the population in Germany.

#### H 2

Opinion polarisation on economic liberalism, the EU, immigration, and the environment has increased over time in Germany.

### Group-Level Opinion Polarisation

So far, we have conceptualised conflict at the societal level by operationalising issue salience and opinion polarisation within the entire population. However, as discussed in the introductory paper of this special issue, social integration—and in our case, social conflict—can be measured not only at societal and individual levels but also at the group level. Groups sharing particular sociodemographic characteristics might hold antagonistic opinions on a set of issues. If such an opinion divide along sociodemographic characteristics is consistent for a set of issues related to the same overarching conflict, this would provide evidence for polarised opinion at the group level. Such group-level opinion polarisation, in turn, relates to the structural component of a cleavage, as described by Bartolini and Mair (1990): The fact that sociodemographic characteristics align with antagonistic opinions on a common set of issues implies that these sociodemographic characteristics structure the population on this conflict. According to our longitudinal perspective on conflict, we would be able to speak of group-level polarisation on globalisation-related issues (1) if we observed systematic sociodemographic differences in opinion on globalisation-related issues and (2) if these differences increased over time, as globalisation pressures have grown in the last decades.

Drawing on the cleavage literature, education is said to be the main factor structuring this conflict around globalisation issues (Bornschier 2018; Hooghe and Marks 2018). Human capital provides citizens with the necessary specialized skills to live an economically secure life in a world of open markets and growing international trade (Teney 2016). Besides education, further sociodemographic characteristics shown to be significantly associated with our globalisation-related issues are gender, income, age, level of urbanisation of place of residence, and region of residence (old vs. new federal states of Germany) (Mau et al. 2020; Teney et al. 2014). Accordingly, we would find evidence of group-level opinion polarisation on globalisation-related issues if the association of these sociodemographic characteristics with attitudinal items on economic liberalism, the EU, immigration, and the environment has increased over time in Germany. This leads us to formulate our last hypothesis:

#### H 3

The association of gender, education, income, age, level of urbanisation of place of residence, and region of residence with opinions favouring economic liberalism, the EU, immigration and the environment have increased over time in Germany.

## Data and Methods

We used the Politbarometer trend data file (Forschungsgruppe Wahlen 2022) for analysing the salience of globalisation-related issues among Germans. For assessing opinion polarisation at both the societal and group levels, we used the cumulated German General Social Survey (GESIS—Leibniz Institute for the Social Sciences 2021) or ALLBUS data. In the following section, we describe these two data sets and our operationalisation of issue salience and opinion polarisation.

### The Politbarometer Trend Data

On a monthly basis, the Politbarometer asks a cross-sectional random sample of German respondents the following open question: “In your opinion, what are the most important and the second most important problems faced by Germany nowadays?”^3^ For our paper, we analysed the answers to this question from May 1989 to December 2020. The average number of respondents to this question was 2474 per month. Respondents provided an open answer to this question without having been presented with any predefined answer categories. Interviewers subsequently categorised respondents’ answers using a predefined coding scheme. This coding scheme remained constant over time. New categories were introduced only when respondents referred to a societal problem that had not been categorised adequately by the current coding scheme. Based on all answers given until the end of 2020, this coding scheme now encompasses 134 categories. We then recoded these 134 categories into 15 issue domains following the coding scheme of Hutter and Kriesi (2019), as presented in Table 1. We consider four out of the 15 categories to be globalisation-related: Economic liberalism regroups issues linked to national economic protectionism, global markets, deregulation, privatisation, and reduction of the national deficit and taxes. The environment category encompasses answers dealing with environment protection, environmental disasters, climate change, and renewable and nuclear energy. The Europe category refers to the EU integration process, EU enlargement, introduction of the euro, and the euro crisis. Lastly, the immigration category encompasses answers relating to asylum seekers, so-called late repatriates, refugees, foreigners, and double citizenship.

**Table 1:** Aggregated issue domains of societal problems in the Politbarometer trend data and their average salience

Globalisation-related issues (%)		Issues not related to globalisation (%)	
Immigration Environment Economic liberalism Europe	9.4 5.3 2.9 2.6	Economic situation Welfare (including health) Security and international conflicts Education Other Democratic renewal Reunification Radicalism and Islamism Cultural liberalism Politics general Infrastructure	34.2 9.7 5.3 4.3 4.0 3.6 3.3 1.8 1.1 1.1 0.8

We then collapsed the individual-level data set, yielding wave-specific voting shares for these 15 issue domains. Since every interviewee had the chance to name two important problems, we calculated every domain’s voting share as the total number of votes falling into that domain divided by the total number of respondents$\times 2$. The resulting share indicates the wave-specific percentage of respondents stating that a problem belonging to this domain is either the single or second most important problem in Germany. We used this share as a measure for the overall societal salience of an issue.

The data that we used in this part of our analysis comprise 371 waves spanning a period of 30 years from 1989 to 2020. Since April 1990, the Politbarometer sample has also covered the population living in the former East Germany. However, for our analysis we excluded all former East German respondents until February 1999 because an appropriate weighting factor has existed only since March 1999. Until the data for February 1999, our sample consists of western German observations weighted by a factor (*v*78) that ensures demographic representability for western Germany. For March 1999 and onwards, our sample consists of both western and eastern German observations weighted with a joint weighting factor (*V*81) that compensates for the oversampling of former East Germans and ensures demographic representability for Germany as a whole.

### The Cumulated ALLBUS Data

We used the cumulated ALLBUS data that cover the period 1991–2018 for assessing opinion polarisation. We selected items that were as close as possible to the issue domains used in the salience analysis. We recoded some of them in such a way that higher values always denoted attitudes more in favour of globalisation. Moreover, we restricted our analysis to items asked more than once in order to be able to capture the process of polarisation. Table 2 presents a list of the selected items. For the EU issue, we used an item on trust in the European Commission and an item on European identification. Unfortunately, better measurements of attitudes towards the EU or EU integration were not available in the cumulated ALLBUS trend survey data. We included both western German and eastern German observations in our sample, weighted by a weight (wghtptew) that ensures demographic representability and accounts for the oversampling of former East Germans.

**Table 2:** Description of the selected ALLBUS items on globalisation-related issues

Related issue domain in Politbarometer	ALLBUS item	Label	Number of answer categories	Original variable name in ALLBUS
EU	Eu_trust	How much do you trust the European Commission?	7^a^	pt19
–	Eu_id	How strongly do you identify with the European community and its population?	4^b^	pn17
Immigration	Mig_cult	The foreigners who live in Germany enrich the cultural life of Germany	7^a^	mp03
–	Mig_job	The foreigners who live in Germany take jobs away from Germans	7^a^	mp06
Economic liberalism	Econlib	Opening up global markets even more will be to everyone’s benefit	5^c^	pa18
Environment	Envir	Stronger measures should be taken to protect the environment	5^c^	pa11

^a^For the bar charts presented in Fig. 2, we aggregated the answer categories 1–2 and 6–7 as the two polar edges

^b^Here, we recoded answer categories 2–3 as the middle category. Categories 1 and 4 constitute the polar edges in the corresponding bar chart in Fig. 2

^c^The bar charts of these items presented in Fig. 2 show answer categories 1 and 5 as polar edges

We analysed opinion polarisation in three parts. First, we depicted the evolution of the opinion distribution over time using bar charts. In order to facilitate the readability of these bar charts, we summarised all answer categories into three categories: two polar edges and a broad middle category containing all nonextreme answer categories (see Table 2 for more details).

Second, as a measure of polarisation, we calculated the agreement index proposed by van der Eijk (2001) for each item and data wave. This index can be calculated for ordinal-scaled items and refers to the proportion of respondents in contiguous answer categories. Applied to the example of a five-point Likert scale, high agreement implies that a majority of cases are found in contiguous answer categories, for example the first and second ones. High disagreement, by contrast, implies that observations fall into noncontiguous answer categories—for instance, 30% in the first answer category, 30% in the third answer category, and 40% in the fifth answer category. Appendix B provides a more detailed description of its computation. The agreement index ranges from −1 to 1. A value of −1 denotes complete disagreement as given by a bimodal distribution where 50% of all respondents fall into the first and the other 50% into the last answer category. A value of 1 refers to complete agreement, where all observations are found in a single category (perfect unimodality). An index value of 0 refers to a uniform distribution, where each answer category realises equally frequent. If the agreement index for any globalisation item falls over time, this constitutes another piece of evidence on polarisation and an emerging social conflict around globalisation (see also Lux and Gülzau 2022 for a similar application). To complement van der Eijk’s agreement index as polarisation indicator, we provide the variance, kurtosis, and skewness of these items in Tables 5–7 in the Appendix.

Third, we analysed group-specific opinion differences on globalisation by running simple linear regressions. In separate regression models, we regressed our six attitudinal items on a list of sociodemographic characteristics, survey year dummies, and their interaction terms. We considered the following sociodemographic characteristics: gender, age (categorised as 18–39 years, 40–59 years, and 60+ years), education (low, middle, high), log personal net income per month, region (eastern or western Germany), and level of urbanisation of the place of residence (< 5000 inhabitants, 5000–50,000 inhabitants, > 50,000 inhabitants). The interaction terms of these sociodemographic variables with the survey year dummies allowed us to investigate our third hypothesis on an increase in group-level opinion polarisation. In the case of missing information regarding the relevant variables, observations were deleted listwise. Table 8 in the Appendix presents the corresponding descriptive statistics.

## Results

We first present the results derived from the Politbarometer data regarding the evolution of salience of globalisation-related issues. Then we discuss the results on opinion polarisation derived from the ALLBUS data.

### Issue Salience

**Fig. 1:**
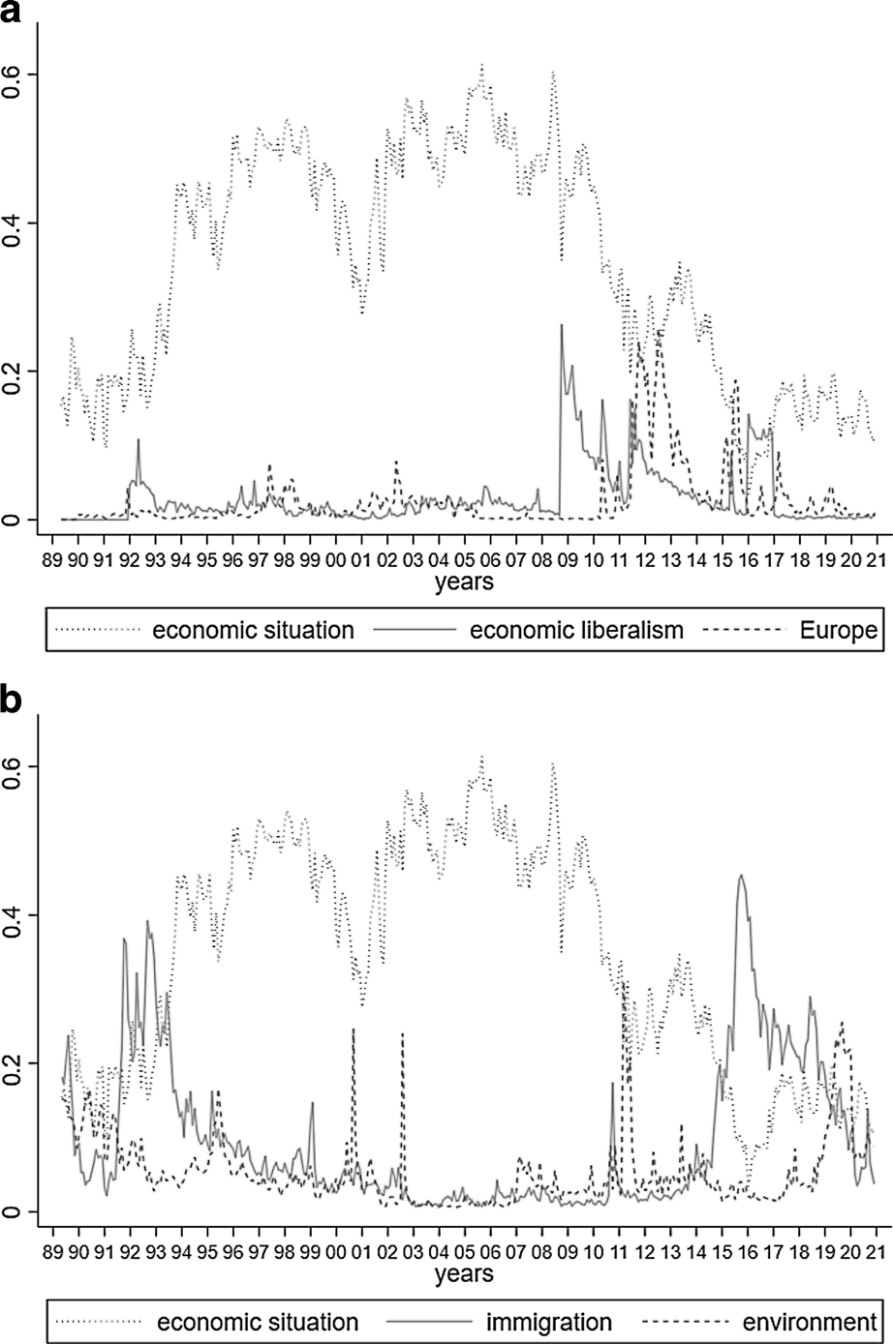
Share of mentions of the two most important problems in Germany, May 1989 through December 2020. The figure is based on the sample of the Politbarometer data for western Germany until February 1999 and on its western and eastern German samples from March 1999 onwards

Figure 1 presents our measure of salience for the four globalisation-related issue domains based on the Politbarometer data for the period 1989–2020. In order to facilitate the interpretation of our relative salience measure, we also depict the most frequently mentioned issue domain, which was the economic situation. Between 1994 and 2010, 40% to 60% of respondents considered a problem belonging to this domain to be one of the main problems in Germany. After 2010, its popularity declined; by the end of 2020, only 11.4% of the Politbarometer respondents considered the economic situation to be one of the two most important problems in Germany.

Figure 1a shows that, compared with the most popular category, economic situation, respondents almost never mentioned economic liberalism until 2008, and until 2011 they almost never mentioned Europe. The sharp increase in the salience of economic liberalism between 2008 and 2012 corresponds to the start of the international financial and banking crisis. In a similar vein, the problem category of Europe experienced popularity peaks related only to critical events, such as the European sovereign debt crisis (the so-called euro crisis) with the EU bailout of Greece, Spain, and Italy between 2011 and 2014. As soon as these critical events lost significance, for instance because they had received a political response, the issue domains of economic liberalism and Europe lost their popularity entirely among Politbarometer respondents. In periods without any critical events, respondents barely mentioned problems belonging to these issue domains.

Turning now to Fig. 1b, which compares the categories of immigration and environment with that of the economic situation, we observe similar popularity peaks associated with critical events for the environment category. For instance, the popularity peak in 2011 relates to the Japanese nuclear power plant disaster in Fukushima, while the popularity peak in 2000 relates to the implementation of ecological taxes in Germany. Between 2017 and the middle of 2019, we observe a tremendous increase in the salience of the environment, which relates to the emission value scandal in the German automotive industry, the United Nations climate summit in Bonn, and, especially, the start of the mobilisation of the Fridays for Future movement. By the end of 2019, however, the environment issue domain had lost popularity. Yet throughout 2020, its salience remained at a high and relatively constant level. As the Politbarometer data currently available cover only the period until the end of 2020, we cannot say how the recent and highly mediatised environmental disasters (e.g., the Ahr Valley flood in 2021 and the recurrent extreme droughts and heat waves) have affected the salience of the environment issue.

Similarly, the issue domain of immigration has gained durable popularity in recent years. At the beginning of the 1990s, Politbarometer respondents mentioned immigration relatively frequently as one of the most important problems in Germany. This period covers not only the reunification process and a series of serious attacks against asylum seekers in Germany but also the ethnic armed conflicts happening in the former Yugoslavia, which resulted in a large number of asylum seekers from the Balkan region in Germany.^4^ Between the mid-1990s and 2015, the immigration problem category lost its relative popularity, with the exception of two short peaks in late 1998 and 2010. By contrast, in 2015 almost every second Politbarometer respondent mentioned a problem belonging to the domain of immigration as being one of the two most important problems in Germany. This sudden gain in salience corresponds to the 2015 European “migrant crisis” (Eurostat 2016). At that time, immigration even trumped the category of economic situation in relative popularity, and from 2015 to 2019, it constituted the most salient problem category. However, compared with its 2015 peak, the immigration issue domain has lost popularity again. Nevertheless, respondents continue to frequently mention problems belonging to the domain of immigration.

All in all, based on our descriptive analysis of the Politbarometer trend data, we have observed an overall increase in salience for the issue of immigration only since 2015 and for the issue of the environment only since 2017. Although the salience of both issues has declined again, immigration and the environment still belonged to the most popular problems mentioned by respondents until the end of the currently available Politbarometer trend data. The loss in salience in 2020 is largely due to the rise in salience of another globalisation-related issue: the COVID-19 pandemic, which became the most salient issue in February 2020 until the end of the available Politbarometer trend data. As two of the four globalisation-related issue domains (i.e., economic liberalism and EU) experienced no durable increase in salience at all, we have to reject our first hypothesis at least in parts. Future waves will reveal whether immigration and the environment gained durable salience or not.

### Opinion Polarisation

**Fig. 2:**
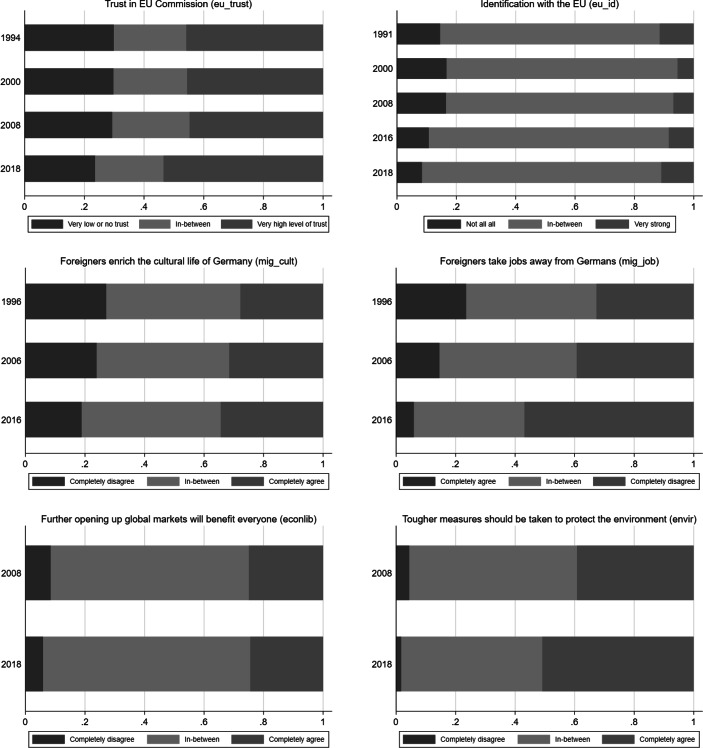
Distribution of the globalisation-related items from the ALLBUS cumulative trend data

We now go on and investigate our further hypotheses on opinion polarisation based on the ALLBUS data. Figure 2 presents the evolution of the opinion distribution of the selected ALLBUS items over time. The first two bar charts in Fig. 2 refer to the items measuring trust in the European Commission and in European identification, respectively. For both EU items, the size of the extreme category encompassing anti-EU attitudes decreased slightly from 2008. By contrast, the size of the other polar edge category constituted of pro-EU attitudes increased slightly during the same period. Thus, because neither polar edge grew in size over time, we cannot speak of an increasing polarisation of attitudes towards the EU among the German population since the 1990s.

Turning to the two bar charts on immigration in the second row, we observe that the size of the categories comprising the most positive attitudes towards immigration has largely increased over time. From 1996 to 2016, the proportion of respondents holding extremely positive attitudes towards immigration grew by 24.2 percentage points in the economic dimension and by 6.6 percentage points in the cultural dimension. This goes hand in hand with a strong decrease in the proportion of citizens with extremely negative attitudes towards immigration. Moreover, the size of the middle-range opinion category remained stable over time. Thus, we observe an overall shift towards more pro-immigrant attitudes in both the cultural and economic dimensions since 1996, rather than an increase in opinion polarisation (see also Lux and Gülzau 2022 for similar results on anti-immigrant attitudes among the German population).

Finally, the items referring to economic liberalism and environment protection were asked in only two ALLBUS waves, in 2008 and 2018. Unfortunately, this means that the assessment of opinion polarisation regarding economic liberalism and environment protection suffers from severe limitations. The bar chart on the left in the last row of Fig. 2 shows the distribution evolution of the item referring to economic liberalism, which points to stable opinions over time because the sizes of the two polar edges remain relatively similar. The bar chart on the right presents the evolution of the opinion distribution on environmental protection measures, showing a general shift towards the support of tougher measures. Here, both the middle category and the adverse polar category decrease in size. It is also noteworthy that, in general, only very few respondents showed extremely negative attitudes towards environment protection measures (4.4% in 2008 and 1.8% in 2018).

Overall, this first part of our investigation of overall opinion polarisation does not provide any support to our second hypothesis: We do not find simultaneous increases in both polar categories of the opinion distribution of any item related to globalisation. In the case of economic liberalism and the EU, attitudes appear relatively stable among the German population. Concerning immigration and environment protection, we detect general opinion shifts towards more supportive attitudes.

Turning to the second part of our analysis on opinion polarisation, Table 3 presents the agreement index of van der Eijk (2001) calculated for each item and each data wave. This calculation is now again based on the original number of answer categories (Table 2). In order to confirm our hypothesis on opinion polarisation, the values on the agreement index for each item should get closer to −1 over time. However, the values either increase over time or remain stable (the latter is the case only for the EU trust item). Thus, these results point towards growing agreement rather than growing disagreement over time. This leads us to reject our second hypothesis: Opinion polarisation on the issues of environment, economic liberalism, immigration, and the EU has not increased over time among the German population.

**Table 3:** Van der Eijk’s agreement index for the ALLBUS items on globalisation-related issues

Item	Label	1991	1994	1996	2000	2006	2008	2016	2018
Eu_trust	Trust in European Commission	–	0.40	–	0.41	–	0.40	–	0.42
Eu_id	Identification with EU	0.35	–	–	0.48	–	0.43	0.43	0.42
Mig_cult	Foreigners enrich cultural life in Germany	–	–	0.07	–	0.15	–	0.18	–
Mig_job	Foreigners take jobs from Germans	–	–	0.01	–	0.15	–	0.44	–
Econlib	Opening world markets benefits everyone	–	–	–	–	–	0.18	–	0.30
Envir	Tougher environmental measures	–	–	–	–	–	0.47	–	0.63

The agreement index ranges from −1 (complete disagreement or perfect bimodality) to 1 (complete agreement or perfect unimodality). A value of 0 denotes a uniform distribution

Last, we investigated the extent to which group-level opinion polarisation increased over time. For this purpose, we estimated a linear regression model by ordinary least squares. Each attitudinal globalisation-related item was regressed on survey year dummies, the sociodemographic characteristics mentioned in the data section, and the interaction terms between them (Table 4). Each column corresponds to one of our six globalisation-related issues as the dependent variable. We used the first year in which the respective globalisation-related item was included in the ALLBUS data as the reference category in our regressions. If we were to observe an increase in group-level polarisation over time, the regression interaction terms between survey years and the sociodemographic characteristics would be significant and would go in the same direction as the main effect of the corresponding sociodemographic characteristic. This would indeed imply that the gap between the sociodemographic characteristic that serves as the reference category and the sociodemographic characteristic measured by the interaction term increased over time. Due to space constraints, we limit our interpretation to consistent trend patterns in order to evaluate our third hypothesis.

**Table 4:** Ordinary least squares regression analysis of six globalisation-related items from ALLBUS trend data

	(1)	(2)	(3)	(4)	(5)	(6)
	Eu_trust	Eu_id	Mig_cult	Mig_job	Econlib	Envir
Label	Trust in European Commission	Identification with EU	Foreigners enrich cultural life	Foreigners take jobs away	Opening world markets	Tougher environmental measures
Baseline year	1994	1991	1996	1996	2008	2008
Further survey years	2000, 2008, 2018	2000, 2008, 2016, 2018	2006, 2016	2006, 2016	2018	2018
*Years*						
Year 2000	0.11	−0.70**	–	–	–	–
	(0.59)	(0.31)	–	–	–	–
Year 2006	–	–	0.76	−0.56	–	–
	–	–	(0.57)	(0.55)	–	–
Year 2008	0.72	−0.64**	–	–	–	–
	(0.47)	(0.26)	–	–	–	–
Year 2016	–	−0.60**	1.15**	1.49***	–	–
	–	(0.26)	(0.55)	(0.53)	–	–
Year 2018	−0.45	−0.78***	–	–	−0.06	0.62**
	(0.48)	(0.27)	–	–	(0.37)	(0.30)
*Male*						
Male	−0.22***	0.12***	0.07	0.16**	0.13**	−0.23***
	(0.07)	(0.04)	(0.08)	(0.08)	(0.05)	(0.04)
Male # Year 2000	0.11	−0.13**	–	–	–	–
	(0.11)	(0.06)	–	–	–	–
Male # Year 2006	–	–	0.03	0.03	–	–
	–	–	(0.11)	(0.10)	–	–
Male # Year 2008	0.24**	−0.10**	–	–	–	–
	(0.09)	(0.05)	–	–	–	–
Male # Year 2016	–	−0.13***	−0.13	−0.07	–	–
	–	(0.05)	(0.10)	(0.10)	–	–
Male # Year 2018	−0.07	−0.18***	–	–	−0.06	−0.02
	(0.09)	(0.05)	–	–	(0.07)	(0.06)
*Age*						
18–39 years	0.02	−0.30***	0.70***	0.67***	−0.13**	0.15***
	(0.09)	(0.05)	(0.10)	(0.10)	(0.07)	(0.05)
40–59 years	−0.08	−0.10**	0.53***	0.37***	−0.26***	0.08
	(0.09)	(0.05)	(0.10)	(0.09)	(0.06)	(0.05)
18–39 years # Year 2000	0.15	0.22***	–	–	–	–
	(0.14)	(0.07)	–	–	–	–
18–39 years # Year 2006	–	–	−0.40***	−0.49***	–	–
	–	–	(0.14)	(0.13)	–	–
18–39 years # Year 2008	0.29**	0.19***	–	–	–	–
	(0.12)	(0.06)	–	–	–	–
18–39 years # Year 2016	–	0.18***	−0.49***	−0.60***	–	–
	–	(0.06)	(0.13)	(0.13)	–	–
18–39 years # Year 2018	0.24**	0.15**	–	–	−0.16*	−0.09
	(0.11)	(0.06)	–	–	(0.09)	(0.07)
40–59 years # Year 2000	0.02	0.06	–	–	–	–
	(0.13)	(0.07)	–	–	–	–
40–59 years # Year 2006	–	–	−0.22*	−0.07	–	–
	–	–	(0.13)	(0.13)	–	–
40–59 years # Year 2008	0.08	0.00	–	–	–	–
	(0.11)	(0.06)	–	–	–	–
40–59 years # Year 2016	–	−0.05	−0.25**	−0.39***	–	–
	–	(0.06)	(0.12)	(0.12)	–	–
40–59 years # Year 2018	0.04	−0.02	–	–	−0.07	−0.03
	(0.11)	(0.06)	–	–	(0.08)	(0.07)
*Education*						
Medium level (“mittlere Reife”)	0.01	0.15***	0.33***	0.36***	−0.14**	−0.11**
	(0.08)	(0.04)	(0.09)	(0.09)	(0.06)	(0.05)
High level (“[Fach‑]Hochschulreife”)	−0.02	0.25***	0.85***	0.99***	−0.35***	0.04
	(0.09)	(0.05)	(0.10)	(0.10)	(0.07)	(0.05)
Medium level # Year 2000	−0.02	−0.04	–	–	–	–
	(0.13)	(0.07)	–	–	–	–
Medium level # Year 2006	–	–	−0.04	−0.04	–	–
	–	–	(0.13)	(0.12)	–	–
Medium level # Year 2008	0.16	0.07	–	–	–	–
	(0.11)	(0.06)	–	–	–	–
Medium level # Year 2016	–	0.04	−0.04	0.23*	–	–
	–	(0.06)	(0.13)	(0.12)	–	–
Medium level # Year 2018	0.10	0.07	–	–	0.19**	0.12
	(0.11)	(0.06)	–	–	(0.09)	(0.07)
High level # Year 2000	0.00	−0.04	–	–	–	–
	(0.14)	(0.07)	–	–	–	–
High level # Year 2006	–	–	−0.01	−0.02	–	–
	–	–	(0.14)	(0.13)	–	–
High level # Year 2008	0.25**	0.17***	–	–	–	–
	(0.11)	(0.06)	–	–	–	–
High Level # Year 2016	–	0.18***	0.19	0.00	–	–
	–	(0.06)	(0.13)	(0.13)	–	–
High level # Year 2018	0.35***	0.26***	–	–	0.35***	−0.01
	(0.11)	(0.06)	–	–	(0.09)	(0.08)
*Log(income)*						
Log(income)	0.05	0.02	0.10*	0.13**	−0.05	−0.06**
	(0.06)	(0.03)	(0.06)	(0.06)	(0.04)	(0.03)
Log(income) # Year 2000	−0.03	0.07	–	–	–	–
	(0.09)	(0.05)	–	–	–	–
Log(income) # Year 2006	–	–	−0.02	0.14*	–	–
	–	–	(0.08)	(0.08)	–	–
Log(income) # Year 2008	−0.17**	0.06	–	–	–	–
	(0.07)	(0.04)	–	–	–	–
Log(income) # Year 2016	–	0.07*	−0.06	−0.04	–	–
	–	(0.04)	(0.08)	(0.08)	–	–
Log(income) # Year 2018	0.05	0.10**	–	–	0.03	−0.02
	(0.07)	(0.04)	–	–	(0.05)	(0.04)
*East*						
East	−0.08	−0.24***	−0.08	−1.05***	0.08	0.08*
	(0.08)	(0.04)	(0.08)	(0.08)	(0.05)	(0.04)
East # Year 2000	−0.13	−0.07	–	–	–	–
	(0.12)	(0.06)	–	–	–	–
East # Year 2006	–	–	−0.06	0.57***	–	–
	–	–	(0.11)	(0.11)	–	–
East # Year 2008	−0.09	0.02	–	–	–	–
	(0.10)	(0.05)	–	–	–	–
East # Year 2016	–	−0.00	−0.32***	0.78***	–	–
	–	(0.05)	(0.11)	(0.10)	–	–
East # Year 2018	−0.16	−0.00	–	–	−0.06	−0.17***
	(0.10)	(0.05)	–	–	(0.07)	(0.06)
*Municipality size*						
Midsized city (5000–50,000 inhabitants)	0.10	N.A.	0.34***	−0.07	0.08	0.19***
	(0.09)	–	(0.10)	(0.09)	(0.07)	(0.05)
Large city (> 50,000 inhabitants)	0.13	N.A.	0.66***	0.32***	0.11	0.32***
	(0.09)	–	(0.10)	(0.10)	(0.07)	(0.06)
Midsized city # Year 2000	−0.01	N.A.	–	–	–	–
	(0.14)	–	–	–	–	–
Midsized city # Year 2006	–	–	−0.12	0.19	–	–
	–	–	(0.13)	(0.13)	–	–
Midsized city # Year 2008	0.10	N.A.	–	–	–	–
	(0.12)	–	–	–	–	–
Midsized city # Year 2016	–	N.A.	−0.09	0.10	–	–
	–	–	(0.13)	(0.13)	–	–
Midsized city # Year 2018	0.11	N.A.	–	–	−0.07	−0.10
	(0.11)	–	–	–	(0.09)	(0.07)
Large city # Year 2000	−0.10	N.A.	–	–	–	–
	(0.14)	–	–	–	–	–
Large city # Year 2006	–	–	−0.32**	−0.07	–	–
	–	–	(0.14)	(0.14)	–	–
Large city # Year 2008	0.21*	N.A.	–	–	–	–
	(0.12)	–	–	–	–	–
Large city # Year 2016	–	N.A.	−0.13	−0.25*	–	–
	–	–	(0.14)	(0.13)	–	–
Large city # Year 2018	0.12	N.A.	–	–	−0.08	−0.12
	(0.12)	–	–	–	(0.10)	(0.08)
Constant	3.15***	2.36***	2.04***	2.63***	3.87***	4.24***
	(0.37)	(0.21)	(0.41)	(0.40)	(0.25)	(0.21)
Observations	8.459	12.541	8.248	8.247	5.507	5.714
R‑squared	0.036	0.095	0.096	0.190	0.025	0.053
Adjusted R‑squared	0.031	0.092	0.093	0.187	0.022	0.050

The regression model presented in column (2) is the only one that does not consider municipality size, as this information is not provided for West Germany in 1991. Standard errors in parentheses

*p < 0.1, **p < 0.05, ***p < 0.01

Turning to the first dependent variable (eu_trust) presented in Table 4, trust in the EU Commission, group differences became significantly larger over time for the youngest cohort compared with the oldest cohort of respondents. Furthermore, differences between the groups of low-educated and highly educated respondents increased significantly over time. Indeed, respondents with different educational levels did not differ significantly from each other on their level of trust in the EU Commission in 1994. By contrast, the group of highly educated respondents held a significantly higher level of trust in the EU commission than the group of low-educated respondents in 2008 and 2018. The remaining interaction terms are either nonsignificant or do not point to consistent trend patterns. If we now look at the second dependent variable, identification as European (eu_id), we see that educational differences and income differences systematically increased over time: Respondents with a high educational level as well as those with a high income identified more and more strongly as European over time than did low-educated respondents or those with a low income. By contrast, gender and age differences in identification as European significantly decreased over time. While men identified significantly stronger with the EU than women did in 1991 (+0.12 points on a scale from 1 to 4), this effect was close to zero in the following years (for example, in 2000 it was 0.12 − 0.13 = −0.01 points). The other interaction terms were either nonsignificant or did not present any consistent trend. The third and fourth columns of Table 4 present the regression results for the two immigration-related attitudinal items, “foreigners enrich cultural life in Germany” (mig_cult) and “foreigners take jobs from Germans” (mig_job). The interaction terms with the survey years and the sociodemographic variables show similar patterns for both immigration items. First, we do not observe any consistent trend towards an increase in group differences in opinion on these immigration items. Second, age differences significantly decreased over time for both items. However, there is a trend pattern unique to the item “foreigners take jobs from Germans” (mig_job): Differences between eastern and western Germans on this item decreased significantly over time (with eastern Germans having significantly more positive attitudes on this item over time).

Lastly, the interpretation of the results of the two remaining dependent variables is less straightforward. The items “opening world markets benefits everyone” (econlib) and “tougher environmental measures should be taken” (envir) have been measured only twice so far. It is therefore dangerous to speak of any trend when observing significant differences in the regression coefficients between two measurement points. We find only a single interaction term for the regression results of both the “opening of world markets” (econlib) and “tougher environmental restrictions” (envir) items that suggests a significant increase in group differences between 2008 and 2018. Age differences between the youngest and oldest cohorts of respondents significantly increased in 2018 compared with 2008 regarding the item “opening world markets benefits everyone” (econlib); in 2018, respondents belonging to the youngest cohort disagreed significantly more than respondents from the oldest cohort on this item. Several interaction terms for the two items econlib and envir point to a decrease in group differences over time, however. The opinion difference between educational groups on the item “opening world markets benefits everyone” (econlib), which had been significant in 2008, came close to 0 by 2018. Furthermore, while eastern Germans held significantly more positive attitudes towards the item “tougher environmental measures should be taken” (envir) in 2008 at the 10% significance level, they showed significantly more negative attitudes than western Germans on the same item 10 years later. As both items were measured at only two points in time, we cannot draw any conclusion from these particular results.

All in all, we find rather little support for our last hypothesis. We find only a consistent trend in increasing group differences for age on trust in the European Commission and on the item on international trade, as well as for educational levels on the items measuring trust in the European Commission and identification as European. The latter item also shows increasing group differences for income. Other sociodemographic differences in our six attitudinal globalisation-related items either remained stable or even decreased over time.

We tested the sensitivity of our results by including an additional control variable for migration status, operationalised by a dummy indicating being born in Germany. Overall, the results reported in Table 4 remain stable once we control for respondents’ migration status. Moreover, we find the same patterns of significance and effect directions as shown in Table 4. In another set of sensitivity analyses, we used a categorised income variable in order to investigate the validity of our linear modelling of the relation of income on the dependent variables. With the exception of the “trust in European Commission” (eu_trust) item, the association of the log of personal monthly net income with our dependent variables is linear. Appendix A contains the respective tables of our sensitivity analyses.

## Conclusion

In this contribution, we investigate the extent to which the conditions for a new globalisation conflict among the German population are met. Drawing on cleavage theory, we stress the structuring force of social conflicts among the population and highlight their integrative function. We argue that a social conflict on globalisation-related issues manifests through sustainable political mobilisation and that it requires two conditions: First, the population overall should consider globalisation-related issues as salient. Second, these globalisation-related issues should be contested within the population overall and between groups. We operationalise these two conditions as issue salience and overall and between-group opinion polarisation. Furthermore, we hypothesise that issue salience and overall and between-group opinion polarisation on globalisation-related issues should have increased over time as globalisation pressures have increased in the last decades in Europe (Gygli et al. 2019). We focus our analysis on four globalisation-related issue domains: immigration, the EU, economic liberalism, and the environment.

Our analysis of the salience of these issue domains among Germans from 1989 to 2020 points to an overall increase in salience for the issues of immigration since 2015 and the environment since 2018. While both immigration and the environment have recently been losing salience, they nevertheless still belonged to the most popular problems mentioned by Germans until the end of the Politbarometer trend data analysed. By contrast, the salience of the two other globalisation-related problems (i.e., economic liberalism and Europe) remained very low during the period 1989–2020 and exhibited short-duration peaks related to critical events only. This pattern reflects the results from studies on the politicisation of the EU in the media. Hutter et al. (2016, p. 283) conclude, for example, from their mass media content analysis that the politicisation of the EU shows “a process of punctuated politicisation, in which a significant but limited number of singular events produce high levels of political conflict for shorter periods of time.” As only two of the four globalisation-related issue domains experienced an increase in salience, and this occurred only very recently, our results can only partly confirm our first hypothesis of an overall increase in the salience of globalisation-related issues over time. Obviously, analyses based on the upcoming Politbarometer data waves will shed light on the sustainability of the popularity of immigration and the environment as the most important problems among Germans. As our results clearly show, critical events have a great impact on issue salience among the German population. Recent and upcoming critical events, such as the COVID-19 pandemic and extreme weather events, are likely to relegate popular issues to second-order problems or push less popular issues to the rank of first-order problems among the population.

Turning now to overall opinion polarisation, our results indicate rather stable attitudes on globalisation-related issues among the German population over time. We only detect a general opinion shift towards more positive attitudes on immigration and environment protection measures. Thus, we cannot speak of an overall opinion polarisation on globalisation-related issues if we understand opinion polarisation as a process whereby citizens position themselves increasingly on the two polar edges of an attitudinal divide (Barber and McCarty 2015, p. 24). While our analysis shows that Germans hold antagonistic positions on some globalisation-related issues, this opinion divide has remained rather stable since the 1990s.

Lastly, our regression analyses of the main sociodemographic characteristics on six attitudinal globalisation-related items enabled us to investigate between-group polarisation over time. Here again, our results provide only very little support for the idea of an increasing between-group polarisation. Age differences in the level of trust in the European Commission and the level of support for international trade increased consistently and significantly over time. Moreover, highly educated Germans as well as Germans with a high income identified more and more strongly as Europeans over time than did Germans with low educational attainment or income. By contrast, the effects of all other sociodemographic differences yield a rather mixed picture without any consistent trend.

However, our analyses of overall and between-group opinion polarisation suffer from several weaknesses due to data limitations. First, some attitudinal items were characterised by a high number of missing cases for some ALLBUS waves (ranging from 0.28% missing cases for the item “foreigners take jobs away” [mig_job] in 1996 to 55% missing cases for the item “trust in European Commission” [eu_trust] in the year 2000). This, in turn, might alter the robustness of our findings on trends in polarisation. Second, some items used in our analyses constitute only poor measurements of opinion on globalisation-related issues—in particular, the items on the EU and on environment protection. Third, the items on international trade and environment protection were included in only two ALLBUS survey waves. Lastly, the items analysed were not included in the same ALLBUS survey waves. This, in turn, hinders any meaningful between-item comparison of opinion polarisation over time. Moreover, we were not able to assess the extent to which citizens hold consistent opinions on distinct issues related to globalisation. In statistical terms, we were not able to compute correlation coefficients between the attitudinal items and investigate their evolution over time. Thus, while the Politbarometer trend data provide a unique opportunity for investigating issue salience over time by virtue of monthly data collection, general social survey trend data as provided by ALLBUS, for example, enable only an unsatisfactory assessment of opinion polarisation over time, at least with regard to globalisation as done here. It is therefore not feasible to draw any definitive conclusions on the evolution of opinion polarisation on globalisation among Germans on the basis of social survey trend data.

All in all, besides the recently increased popularity of immigration and the environment as the most important problems among Germans, we find little empirical support for the idea of an emerging conflict around globalisation-related issues among the German population. Our results come as a surprise when we consider a common interpretation that receives a great deal of attention in the public debate, such as the rise of a globalisation backlash or a conflict between winners and losers of globalisation in Germany. Such an interpretation tends to be drawn without a longitudinal perspective. As we have shown, the population does hold antagonistic positions on some globalisation-related issues, but this opinion divide has remained constant over time. Moreover, such an interpretation often fails to consider the salience given by the population to such issues. Hence, our study points to the importance of fine-grained and sound descriptive analyses of such a highly mediatised interpretation. This helps contextualise and relativise the somewhat alarmist conclusions attracting a great deal of resonance in the public debate (see Mau et al. 2020 for a similar argument).

Furthermore, our study provides results that contradict the current social science debate on the rise of a globalisation cleavage. Indeed, our results highlight the lack of a structural component in Germany, which would be necessary to be able to speak of a globalisation cleavage as defined by Bartolini and Mair (1990). Thus, the current politicisation and mobilisation around globalisation-related issues in Germany seem to be driven by the supply rather than by the demand side of the political system. These results are in line with one argument of Walter (2021): The electoral success of political actors with an antiglobalisation programme is the result of an increasing mobilisation of existing antiglobalisation attitudes rather than the result of a shift in public opinion towards globalisation-related issues. Indeed, political entrepreneurs offering a political platform for citizens holding antiglobalisation attitudes have flourished in the last decade (Bornschier 2018). By trying to mobilise voters on these issues, they have increased the visibility of globalisation-related issues in the public debate (Walter 2021). The politicisation of particular issues driven by political parties has been shown, in turn, to be associated with greater opinion polarisation among the population on the corresponding issue (Ares 2022). Thus, the political context largely matters in shaping the structural component of a cleavage. In-depth studies on the interaction between the politicisation of public debate and opinion polarisation on globalisation-related issues would help us to better understand the interdependency among all three components of a cleavage.

Furthermore, our results highlight the need for more in-depth empirical studies focusing on the demand side of this assumed globalisation cleavage. Indeed, investigating the extent to which our results can be transposed to other (Western) EU countries would make an important contribution to the academic debate on the rise of a globalisation cleavage. Additionally, fine-grained analyses of trends in opinion polarisation along other key sociodemographic characteristics are essential for a better assessment of a potential rise of social conflicts due to globalisation pressures (see, for instance, Dochow-Sondershaus and Teney (2022) for opinion polarisation along occupational classes). Another important and complementary research avenue concerns the role of the media in (the perception of) a conflict on globalisation-related issues. Indeed, both traditional media (Czymara and Dochow 2018) and social media (Bail et al. 2018) have been shown to play an important role in shaping attitudes towards globalisation-related issues as well as for their perceived salience. Considering traditional and social media as binding links between the supply and demand sides might help us understand the mismatch between the popular perception of a growing opinion polarisation on globalisation-related issues and our contradictory findings.

## Appendix A

**Table A.1:** Variance of the ALLBUS items on globalisation-related issues

Item	Label	1991	1994	1996	2000	2006	2008	2016	2018
Eu_trust	Trust in European Commission	–	2.06	–	1.97	–	2.04	–	1.95
Eu_id	Identification with EU	0.79	–	–	0.62	–	0.67	0.63	0.64
Econlib	Opening world markets benefits everyone	–	–	–	–	–	1.68	–	1.45
Envir	Tougher environmental measures	–	–	–	–	–	1.30	–	0.91
Mig_job	Foreigners take jobs from Germans	–	–	4.15	–	3.53	–	2.58	–
Mig_cult	Foreigners enrich cultural life in Germany	–	–	3.77	–	3.38	–	3.36	–

**Table A.2:** Skewness of the ALLBUS items on globalisation-related issues

item	Label	1991	1994	1996	2000	2006	2008	2016	2018
Eu_trust	Trust in European Commission	–	0.10	–	−0.02	–	0.11	–	−0.13
Eu_id	Identification with EU	0.18	–	–	0.33	–	0.14	0.03	−0.02
Econlib	Opening world markets benefits everyone	–	–	–	–	–	−0.28	–	−0.43
Envir	Tougher environmental measures	–	–	–	–	–	−1.00	–	−1.42
Mig_job	Foreigners take jobs from Germans	–	–	−0.08	–	−0.33	–	−0.90	–
Mig_cult	Foreigners enrich cultural life in Germany	–	–	0.02	–	−0.15	–	−0.24	–

**Table A.3:** Kurtosis of the ALLBUS items on globalisation-related issues

item	Label	1991	1994	1996	2000	2006	2008	2016	2018
Eu_trust	Trust in European Commission	–	2.54	–	2.33	–	2.52	–	2.44
Eu_id	Identification with EU	2.29	–	–	2.74	–	2.45	2.54	2.54
Econlib	Opening world markets benefits everyone	–	–	–	–	–	1.88	–	2.15
Envir	Tougher environmental measures	–	–	–	–	–	3.14	–	4.63
Mig_job	Foreigners take jobs from Germans	–	–	1.79	–	2.07	–	3.12	–
Mig_cult	Foreigners enrich cultural life in Germany	–	–	1.92	–	2.07	–	2.10	–

**Table A.4:** Summary statistics of ALLBUS variables

		Mean	SD	Min	Max	*N*/%
*Dependent variables*						
Eu_trust	Trust in European Commission	3.41	1.42	1	7	10,322
Eu_id	Identification with EU	2.36	0.83	1	4	15,059
Econlib	Opening world markets benefits everyone	3.45	1.25	1	5	6,608
Envir	Tougher environmental measures	4.10	1.06	1	5	6,863
Mig_job	Foreigners take jobs from Germans	4.66	1.92	1	7	10,345
Mig_cult	Foreigners enrich cultural life in Germany	4.10	1.88	1	7	10,340
*Independent variables*						
Male	–	0.49	0.50	0	1	50,013
East	–	0.33	0.47	0	1	50,013
Linc	Log of income	6.94	0.73	0	11	40,312
Age	Age in categories	–	–	1	3	49,939
	1: 18–39 years old	–	–	–	–	34.80%
	2: 40–59 years	–	–	–	–	36.22%
	3: 60 years and older	–	–	–	–	28.98%
Educ	Educational attainment in categories	–	–	1	3	49,639
	1: Low	–	–	–	–	40.34%
	2: Middle	–	–	–	–	32.63%
	3: High	–	–	–	–	27.03%
Munsize	Municipality size in categories	–	–	1	3	48,374
	1: Less than 5000 inhabitants	–	–	–	–	21.43%
	2: 5000 to 50,000 inhabitants	–	–	–	–	41.79%
	3: More than 50,000 inhabitants	–	–	–	–	36.78%

We recoded educational attainment in three categories: “Low” refers to having no school-leaving certificate or elementary school certificate (“Volks- or Hauptschulabschluss”), “middle” refers to having intermediary secondary qualifications (“Mittlere Reife”), and “high” refers to having school-leaving certificates qualifying for studies at the university level (“Fach- und Hochschulreife”)

## Appendix B

The computation of the measure of agreement A (van der Eijk 2001) can be summarised as follows:

- First, decompose the empirical distribution into layers. Each layer consists of the same number of categories as the original distribution, and each category consists of either zero cases or exactly as many cases as the other categories. Therefore, these layers can also be described as semi-uniform components of the original distribution. They are then represented by binary terms, where 0s represent empty categories and 1s represent nonempty ones. Please refer to van der Eijk (2001) for a graphical representation. An index $i=1{,}\ldots {,}K$ makes it possible to differentiate between the various layers.
- Calculate the measure of unimodality *U*_*i*_ for any layer *i* containing both 0s and 1s:

$$U_{i}=\frac{\left(K-2\right)\times TU-\left(K-1\right)\times TDU}{\left(K-2\right)\times \left(TU+TDU\right)}{,}$$

where K is the total number of categories in the rating scale, TU is the number of triples of categories conforming to unimodality, and TDU is the number of triples of categories deviating from a unimodal pattern. If layer *i* consists of 1s only, then $TU=TDU=0$ and $U_{i}=1$.

- Agreement in layer *i* is measured as follows:

$$A_{i}=U_{i}\times \left(1-\frac{\left(S-1\right)}{\left(K-1\right)}\right){,}$$

where S is the number of nonempty categories in this layer, and K is the total number of categories in the rating scale.

- Finally, the layer-specific agreement measures are added as follows:

$$A=\sum _{i}A_{i}\times w_{i}{,}$$

where *w*_*i*_ is layer *i*’s share of observations, serving as a weight.

## Footnotes

1 See, for example, the KOF Globalisation Index for Europe in Gygli et al. (2019).

2 We focus on opinion polarisation and refrain from discussing other types of polarisation that have been receiving much attention in recent years, such as affective polarisation (see Druckman and Levendusky 2019) or partisan polarisation (e.g., Barber and McCarty 2015).

3 The formulation of this question is not an ideal measure of issue salience, as correctly pointed out by Wlezien (2005): Respondents were asked about the most important problem rather than the most important issue. Issue and problem are not perfect synonyms. Respondents might indeed perceive an issue as important but not consider it a problem (for instance, if they are satisfied with the way the government is dealing with the issue). This limitation in the analysed survey item should be borne in mind when interpreting our results on issue salience.

4 Recall, however, that our analysis of the Politbarometer data excludes the population residing in eastern Germany until February 1999.
